# Alternate partial root-zone drip irrigation improves water– and nitrogen– use efficiencies of sweet-waxy maize with nitrogen fertigation

**DOI:** 10.1038/s41598-017-17560-2

**Published:** 2017-12-08

**Authors:** Fengbei Fu, Fusheng Li, Shaozhong Kang

**Affiliations:** 1 College of Agriculture, Guangxi University, Nanning, 530005 China; 2 Guangxi Academician Work Station of The New Technology of Water–saving Agriculture in Karst Region, Nanning, 530005 China; 3 Center for Agricultural Water Research in China, China Agricultural University, Beijing, 100083 China

## Abstract

Alternate partial root–zone drip irrigation (ADI) or fertigation has favorable effect on crop water- and nitrogen- use efficiencies (WUE and NUE). However, the advantage of combined application of ADI and nitrogen fertigation on crop WUE and NUE remains unclear. A pot experiment was conducted to investigate the impact of three irrigation methods (CDI conventional drip irrigation (both halves of pot irrigated), ADI (both halves of pot alternatively irrigated) and FDI fixed partial root–zone drip irrigation (fixed half of pot irrigated)) and five nitrogen treatments (F0 no N supplied, F1-F4 0.2, 0.18, 0.16 and 0.14 g N per kg soil via fertigation) on sweet-waxy maize. Compared with CDI, ADI reduced water consumption by 19.9%, but increased water use efficiency based on dry seed yield (WUEs) by 32.3%, and also enhanced nitrogen apparent recovery fraction (Nrf) and nitrogen agronomic efficiency (NAE). F1-F4 augmented dry mass accumulation, dry seed yield and total nitrogen uptake if compared to F0. Moreover, F2-ADI had higher shoot and total dry masses, WUEs, total nitrogen uptake, Nrf and NAE. Thus ADI increased nitrogen uptake, WUE and NUE of sweet-waxy maize with nitrogen fertigation of 0.18 g N per kg soil in this study.

## Introduction

Agriculture is facing with the shortage of irrigation water, severe drought and low irrigation water use efficiency in China. Previous study showed seasonal water shortage and low water use efficiency in Guangxi, southern China^1^, which leads to search for high efficient water–saving method^2^. Alternate partial root–zone irrigation (APRI) or alternate partial root–zone drip irrigation (ADI) is a water–saving method by supplying alternate wetting and drying cycling to the root-zones, which can reduce irrigation water^3,4^. Researches on different crops, e.g. maize, potato, peach and cotton indicated that APRI or ADI performs well in increasing water use efficiency (WUE) without a greater yield loss^5–8^. Zorica *et al*.^5^ evaluated the effects of full irrigation and partial root drying irrigation (or called fixed partial root-zone irrigation, approximately fixed half of the root system is irrigated normally while the remaining fixed half is drying without irrigation) on nitrogen (N) content in field potatoes, and observed an increase in N content in potato tubers under partial root–zone drying irrigation in comparison of full irrigation. Li *et al*.^8^ reported that APRI increases maize N apparent recovery fraction by 16.4%, suggesting that APRI can improve N use efficiency in comparison of conventional irrigation.

In China, N fertilizer consumption was 1117 million tons in 1987, and increased to 3391 million tons in 2012^9^. The increasing N fertilizer consumption leads to environmental problems such as greenhouse gas emission and N loss. Fertigation, which integrates irrigation and fertilization, has showed many advantages, including direct supplement to root-zone, a lasting, stable environment of water and fertilizer, less soil fixation to fertilizer and less greenhouse gas emission^10,11^. Some studies indicated that fertigation can contribute to higher yield, nutrient uptake^12,13^ and nutrient utilization under rational fertilization level^12^. Bai *et al*.^14^ reported that compared with conventional fertilization, fertigation increases N use efficiency (NUE) by 14.3%. In addition, some studies showed that fertigation can also improve crop WUE^15–18^.

Previous studies showed alternate partial root–zone drip irrigation (ADI) or fertigation has benefit effect on crop dry mass accumulation, yield, N uptake, and water- and nitrogen- use efficiencies (WUE and NUE)^12,18–20^. However, the advantage of combined application of ADI and fertigation on crop yield, N uptake, WUE and NUE remains unresolved, so the hypothesis of this study was that the combined application of alternate drip irrigation and rational N fertigation can increase N uptake, WUE and NUE of sweet-waxy maize. In this study, a pot experiment was conducted to investigate the effect of three drip irrigation methods and five N fertigation strategies on dry mass accumulation, seed yield, N uptake, WUE and NUE of sweet-waxy maize, so as to reveal alternate root–zone drip irrigation combined with rational nitrogen fertigation for sweet-waxy maize production.

## Results

### Dry mass accumulation and dry seed yield

Table 1 shows the effect of irrigation method and nitrogen (N) treatment on dry mass accumulation and dry seed yield of sweet-waxy maize. Irrigation method and N treatment had remarkable impacts on shoot, root and total dry masses (P < 0.05) and their interaction influenced shoot and total dry masses significantly (Table 1).

**Table 1:** Mean dry mass accumulation and dry seed yield of sweet-waxy maize for different irrigation methods and N treatments. Analysis of variance (ANOVA) *P* values were shown (*P* < 0.05, significance; *P* > 0.05, no significance). Different small letters in the same column are significantly different for various N treatments or irrigation methods (*P* < 0.05), and the same letters are not markedly different for various N treatments or irrigation methods (*P* > 0.05). F0: no N supplied; F1: 0.2 g N kg^−1^ soil; F2: 0.18 g N kg^−1^ soil; F3: 0.16 g N kg^−1^ soil; F4: 0.14 g N kg^−1^ soil. CDI: conventional drip irrigation, ADI: alternate partial root–zone drip irrigation, FDI: fixed partial root–zone drip irrigation. The symbols in the following Tables are the same as this Table.

Treatment	Shoot dry mass (g plant^−1^)	Root dry mass (g plant^−1^)	Total dry mass (g plant^−1^)	Dry seed yield (g plant^−1^)
F0	45.73 c	9.66 c	55.40 c	38.53 d
F1	82.10 a	17.58 a	99.68 a	62.48 a
F2	84.85 a	17.34 a	102.19 a	63.80 a
F3	75.90 b	16.21 b	92.11 b	57.09 b
F4	73.03 b	15.39 b	88.42 b	52.19 c
CDI	72.02 a	15.31 a	87.33 a	55.14 a
ADI	77.97 a	16.25 a	94.22 a	58.25 a
FDI	66.97 a	14.15 a	81.12 a	51.07 a
Significance test (*P* values)				
N treatment	<0.001	<0.001	<0.001	<0.001
Irrigation method	<0.001	<0.001	<0.001	<0.001
N treatment × irrigation method	<0.001	0.754	<0.001	0.522

**Figure 1:**
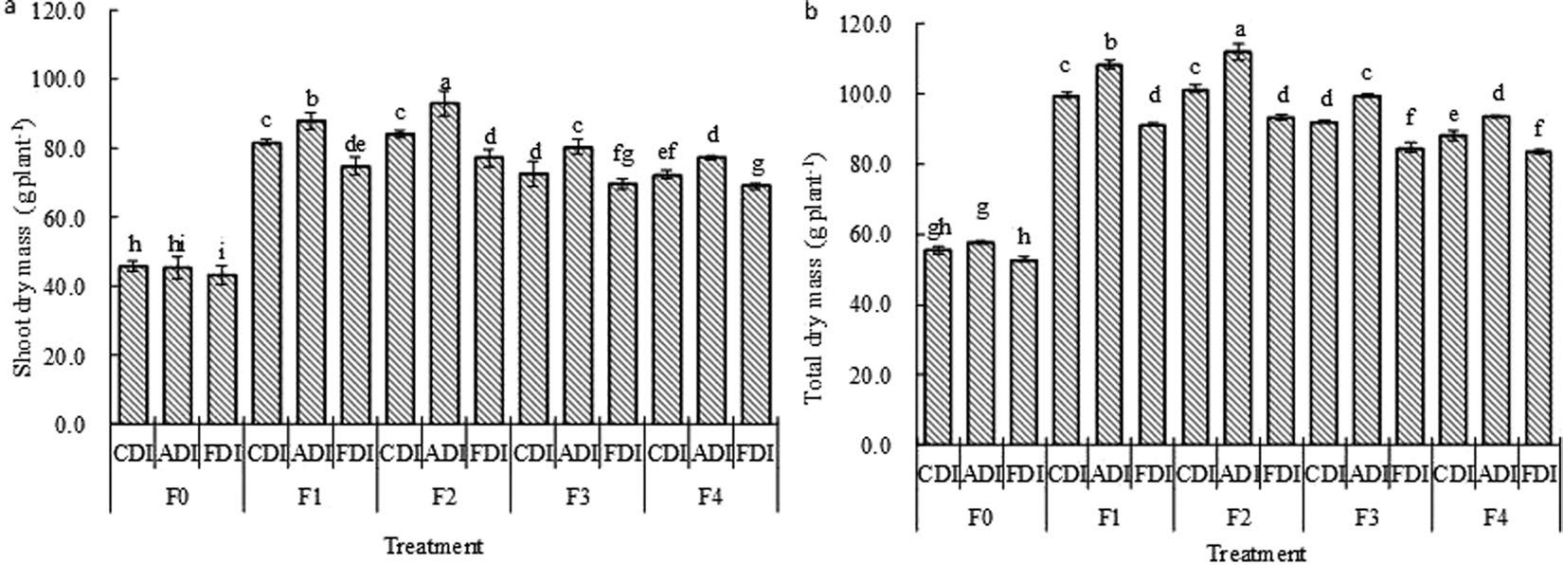
Effects of irrigation method and N treatment on shoot dry mass (**a**) and total dry mass (**b**) of sweet-waxy maize. Data points are averaged values ± standard errors (n = 3), different small letters on the bars are significantly different (*P* < 0.05), and the same letters are not significantly different (*P* > 0.05). F0: no N supplied; F1: 0.2 g N kg^−1^ soil; F2: 0.18 g N kg^−1^ soil; F3: 0.16 g N kg^−1^ soil; F4: 0.14 g N kg^−1^ soil. CDI: conventional drip irrigation, ADI: alternate partial root–zone drip irrigation, FDI: fixed partial root–zone drip irrigation. The symbols in Figs 2 and 3 are the same as Fig. 1.

ADI increased shoot, root and total masses while FDI reduced these indices slightly if compared to CDI. Compared with no N treatment (F0), N fertigation treatments (F1-F4) enhanced shoot, root and total dry masses by 58.1–95.8%, 56.8–86.8% and 57.9–94.1%, respectively (Table 1). Moreover, the highest shoot and total dry masses were observed in F2-ADI (Fig. 1).

Table 1 shows marked effects of irrigation method and N treatment on dry seed yield. Mean dry seed yield in ADI was slightly higher than that in CDI, while mean dry seed in FDI was slightly lower than that in CDI. N fertigation treatments (F1-F4) enhanced mean dry seed yield by 31.4–74.6% if compared with F0, and the differences were notable.

### Water use

**Figure 2:**
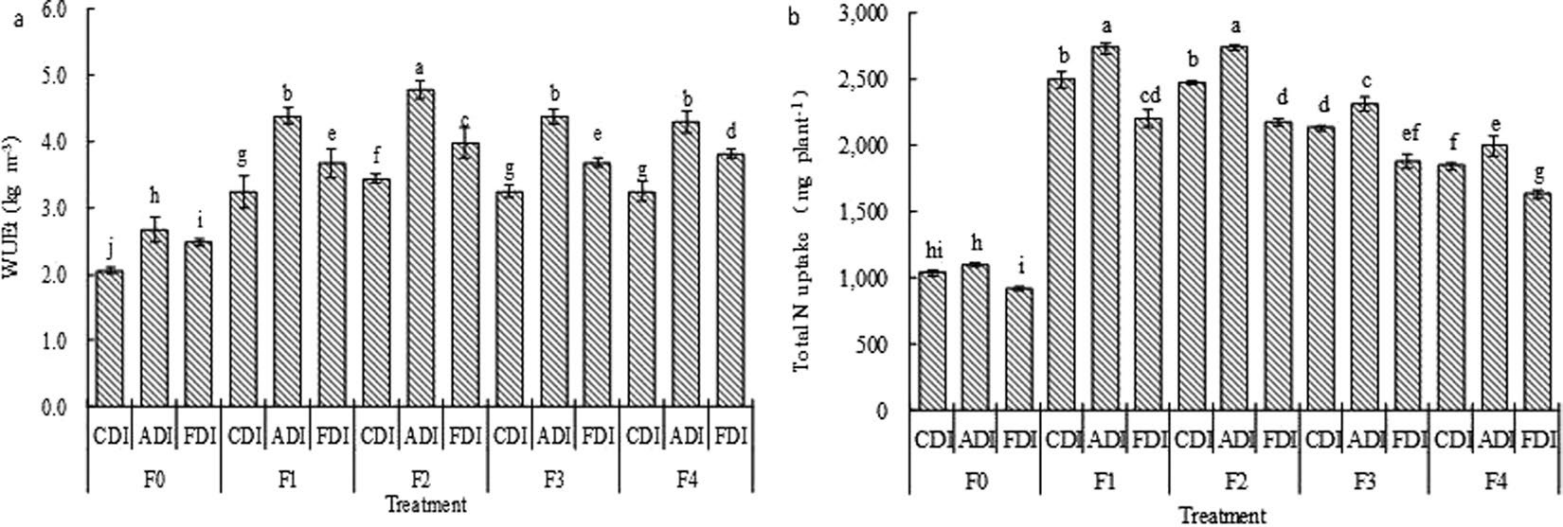
Effects of irrigation method and N treatment on water use efficiency on the basis of total dry mass (WUEt) (**a**) and total N uptake (**b**) of sweet-waxy maize. Data points are averaged values ± standard errors (n = 3), different small letters on the bars are significantly different (*P* < 0.05), and the same letters are not significantly different (*P* > 0.05).

Table 2 shows the effect of irrigation method and N treatment on water use of sweet-waxy maize. Irrigation method and N treatment remarkably affected water consumption, water use efficiency on the basis of total dry mass (WUEt) or dry seed yield (WUEs), but their interaction has not significant impact on these indices (Table 2). Compared to CDI, ADI and FDI decreased mean water consumption of maize by 19.9% and 19.8%, respectively. Hence, ADI and FDI enhanced WUEt by 34.4% and 15.7%, and WUEs by 32.3% and 15.6%, respectively (Table 2). The WUEt and WUEs was 39.7–79.5% and 28.4–61.5% higher in N fertigation treatments than in no N treatment, respectively, and the difference was obvious. On average, F2-ADI enhanced WUEs by 39.0% if compared with the other treatments (Fig. 2).

**Table 2:** Mean water use of sweet-waxy maize for different irrigation methods and N treatments. WUEt: water use efficiency on the basis of total dry mass, WUEs: water use efficiency on the basis of dry seed yield. Different small letters in the same column are significantly different for various N treatments or irrigation methods (*P* < 0.05), and the same letters are not markedly different for various N treatments or irrigation methods (*P* > 0.05).

Treatment	Water consumption (kg pot^−1^)	WUEt (kg m^−3^)	WUEs (kg m^−3^)
F0	23.36 b	2.40 b	1.67 c
F1	26.75 a	3.77 a	2.36 ab
F2	25.40 ab	4.07 a	2.54 a
F3	24.67 ab	3.77 a	2.34 ab
F4	23.60 b	3.79 a	2.23 b
CDI	28.54 a	3.05 c	1.92 c
ADI	22.86 b	4.10 a	2.54 a
FDI	22.87 b	3.53 b	2.22 b
Significance test (*P* values)			
N treatment	<0.001	<0.001	<0.001
Irrigation method	<0.001	<0.001	<0.001
N treatment × irrigation method	0.666	<0.001	0.151

### N content

Impacts of irrigation method and N treatment on N contents in shoots, roots and seeds of sweet-waxy maize are shown in Table 3. Irrigation method had marked effect on N contents of shoots and seeds, and N treatment influenced the contents of shoots, roots and seeds N significantly, but their interaction did not affect maize N content notably.

**Table 3:** Mean N contents in shoots, roots and seeds of sweet-waxy maize for different irrigation methods and N treatments. *P* values were shown (*P* < 0.05, significance; *P* > 0.05, no significance). Different small letters in the same column are significantly different for various N treatments or irrigation methods (*P* < 0.05), and the same letters are not markedly different for various N treatments or irrigation methods (*P* > 0.05).

Treatment	Shoot N content (g kg^−1^)	Root N content (g kg^−1^)	Seed N content (g kg^−1^)
F0	10.62 d	5.84 c	12.23 c
F1	15.06 a	10.32 a	16.84 a
F2	14.46 ab	10.43 a	16.44 a
F3	14.04 b	9.38 b	15.49 b
F4	12.49c	8.93 b	14.74 b
CDI	13.49 a	9.08 a	15.29 a
ADI	13.62 a	9.14 a	15.70 a
FDI	12.89 a	8.72 a	14.44 a
Significance test (*P* values)			
N treatment	<0.001	<0.001	<0.001
Irrigation method	0.020	0.215	0.001
N treatment × irrigation method	1.000	1.000	0.999

ADI and FDI did not significantly have higher or lower mean N contents in shoots, roots and seeds than CDI. In F0, mean N contents in shoots, roots and seeds were 10.63, 5.84 and 12.23 g kg^−1^, while these values in N fertigation treatments (F1-F4) were raised by 16.5–43.0%, 49.8–81.6% and 19.3–41.9%, respectively, and the differences were remarkable (Table 3).

### N uptake

Table 4 shows the effect of irrigation method and N treatment on N uptake of sweet-waxy maize. Significant effects of irrigation method and N treatment on shoot, root, seed and total N uptakes were obtained, and their interaction only influenced total N uptake notably.

**Table 4:** Mean N uptake of sweet-waxy maize for different irrigation methods and N treatments. Analysis of variance (ANOVA) *P* values were shown (*P* < 0.05, significance; *P* > 0.05, no significance). Different small letters in the same column are significantly different for various N treatments or irrigation methods (*P* < 0.05), and the same letters are not markedly different for various N treatments or irrigation methods (*P* > 0.05).

Treatment	Shoot N uptake (mg plant^−1^)	Root N uptake (mg plant^−1^)	Seed N uptake (mg plant^−1^)	Total N uptake (mg plant^−1^)
F0	486.24 d	56.41 c	472.26 d	1014.91 d
F1	1238.70 a	181.78 a	1054.25 a	2474.73 a
F2	1228.04 a	181.20 a	1049.94 a	2459.18 a
F3	1067.08 b	152.25 b	885.36 b	2104.69 b
F4	912.94 c	137.60 b	771.29 c	1821.84 c
CDI	992.52 a	143.84 a	857.29 ab	1993.65 a
ADI	1086.26 a	153.98 a	930.98 a	2171.22 a
FDI	881.03 a	127.71 a	751.59 b	1760.33 a
Significance test (*P* values)				
N treatment	<0.001	<0.001	<0.001	<0.001
Irrigation method	<0.001	<0.001	<0.001	<0.001
N treatment × irrigation method	0.100	0.885	0.809	0.006

Compared with CDI, ADI increased mean shoot and total N uptakes slightly, but FDI decreased mean seed N uptake slightly. In comparison of F0, N fertigation treatments (F1-F4) increased shoot, root, seed and total N uptakes by 86.4–164.5%, 142.7–228.6%, 59.6–127.1% and 77.8–150.2%, respectively, and the differences were obvious (Table 4). F2-ADI had the highest total N uptake (Fig. 2). On average, total N uptake in F2-ADI was 59.0% higher than that in the other treatments.

### NUEs

**Figure 3:**
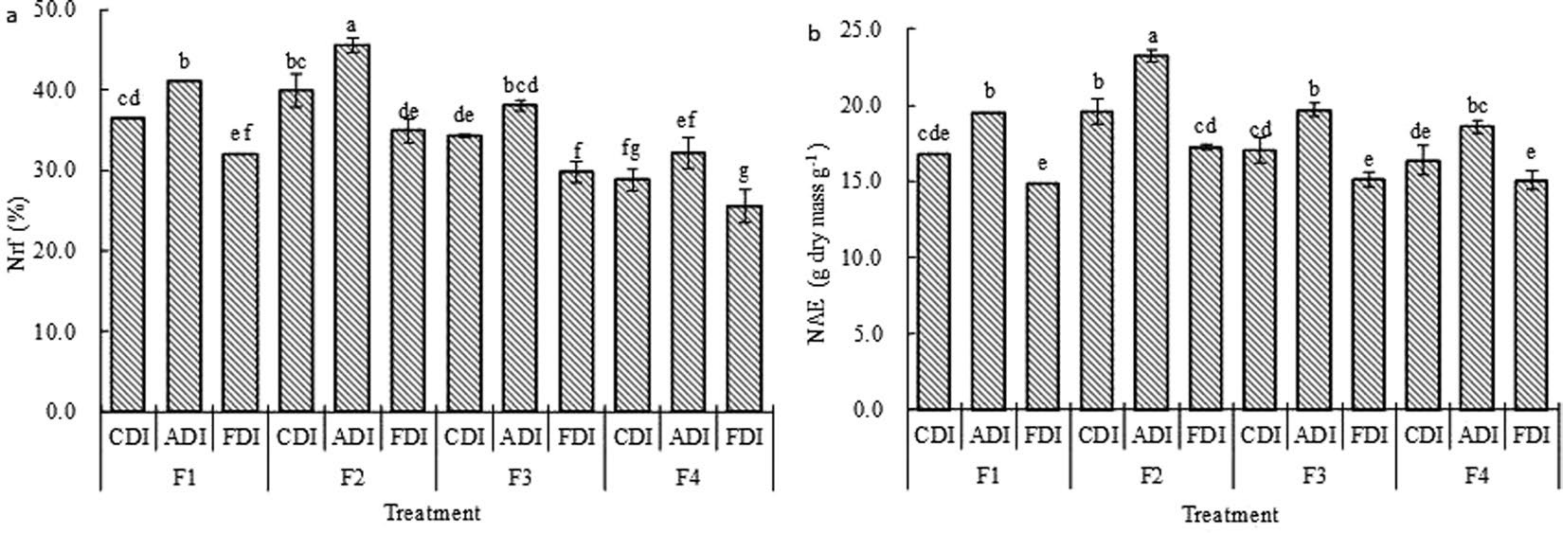
Effects of irrigation method and N treatment on nitrogen apparent recovery fraction (N_rf_) (**a**) and nitrogen agronomic efficiency (NAE) (**b**) of sweet-waxy maize. Data points are averaged values ± standard errors (n = 3), different small letters on the bars are significantly different (*P* < 0.05), and the same letters are not significantly different (*P* > 0.05).

Impacts of irrigation method and N treatment on nitrogen use efficiency of sweet-waxy maize were shown in Table 5. Marked effects of irrigation method, N treatment and their interaction on nitrogen apparent recovery fraction (Nrf) were found. CDI had an average Nrf of 27.9%. ADI increased Nrf by 12.4% and FDI lowered it by 12.3% when compared with CDI. In a certain range of N rates, Nrf enhanced with the rising of N rates, but declined when N rate was over 0.18 g N per kg soil. Moreover, F2-ADI had the highest Nrf (Fig. 3).

**Table 5:** Mean nitrogen apparent recovery fraction (Nrf), nitrogen use efficiency (NUE), nitrogen physiological efficiency (NPE), nitrogen agronomic efficiency (NAE), root nitrogen uptake efficiency (NRE) of sweet-waxy maize for different irrigation methods and N treatments. Analysis of variance (ANOVA) *P* values were shown (*P* < 0.05, significance; *P* > 0.05, no significance). Different small letters in the same column are significantly different for various N treatments or irrigation methods (*P* < 0.05), and the same letters are not markedly different for various N treatments or irrigation methods (*P* > 0.05).

Treatment	Nrf (%)	NUE (g dry mass g^−1^ N)	NPE (g dry mass g^−1^ N)	NAE (g dry mass g^−1^ N)	NRE (mg shoot N g^−1^ root DM)
F0	—	92.84 a	—	—	99.62 d
F1	36.50 ab	65.73 d	46.78 c	17.06 b	130.23 a
F2	40.12 a	67.64 d	49.83 b	20.02 a	131.46 a
F3	34.06 b	71.11 c	50.88 b	17.27 b	120.40 b
F4	28.82 c	77.44 b	58.02 a	16.68 b	109.36 c
CDI	27.89 b	74.07 a	40.27 a	13.94 b	119.08 a
ADI	31.34 a	72.75 a	41.77 a	16.20 a	122.01 a
FDI	24.46 c	78.02 a	41.25 a	12.47 c	113.55 a
Significance test (P values)					
N treatment	<0.001	<0.001	<0.001	<0.001	<0.001
Irrigation method	<0.001	<0.001	0.395	<0.001	0.010
N treatment × irrigation method	0.044	0.738	0.997	0.005	1.000

Significant effects of irrigation method and N treatment on nitrogen use efficiency (NUE) were observed, but there was no remarkable effect of their interaction on NUE (Table 5). As a whole, mean NUE decreased with the raise of N rates.

There was notable effect of N treatment on nitrogen physiological efficiency (NPE), but no marked effect of irrigation method on NPE. Similarly, the interaction of irrigation method and N treatment did not significantly impact NPE (Table 5). In summary, mean NPE also reduced with the rising of N rates.

Remarkable effects of irrigation method, N treatment and their interaction on nitrogen agronomic efficiency (NAE) were found (Table 5). Compared to CDI, ADI increased mean NAE by 16.2%, while FDI decreased it by 10.6%. In a certain range of N rates, NAE enhanced with the increasing of N rates, but decreased when N rate was over 0.18 g N per kg soil. F2-ADI had the highest NAE, and was 9.2–38.5% higher than the other treatments (Fig. 3).

As shown in Table 5, irrigation method and N treatment showed obvious effects on root nitrogen uptake efficiency (NRE), but their interaction did not affect NRE markedly. Mean NRE in ADI was 122.0 mg shoot N g^−1^ root, higher than that in CDI. But mean NRE in FDI was 113.6 mg shoot N g^−1^ root, slightly lower than that in CDI. In a certain range of N rates, NRE rose with the raise of N rates, but declined when N rate was over 0.18 g N per kg soil.

## Discussion

Earlier studies showed that alternate partial root–zone irrigation (APRI) or alternate partial root–zone drip irrigation (ADI) decreases crop dry mass accumulation and raises water use efficiency (WUE) in comparison of conventional irrigation (no alternating drying–wetting irrigation) or conventional drip irrigation (CDI)^6–8,21^. The results of this study showed that ADI enhanced dry mass accumulation slightly and water use efficiency on the basis of total dry mass (WUEt) of sweet-waxy maize greatly if compared with CDI. Nong *et al*.^22^ showed that APRI from seedling to jointing stages of maize has no obvious effect on total dry mass and increases WUEt notably. Our study further indicated that ADI influenced maize seed yield with N fertigation, and both ADI and FDI decreased maize water consumption, thus raised maize water use efficiency on the basis of dry seed yield (WUEs) if compared to CDI, which was similar to the results of the previous studies^23–25^. Overall, the highest WUEt and WUEs were observed in ADI. This result can be attributed to the characteristics of ADI, which forms alternate drying and wetting circles in root system, and enhances abscicic acid in the roots under drought stress and causes stomata closure, thereby leads to the reduction in transpiration loss and the improvement in crop WUE without much yield decrease^3,4^.

Previous studies also reported that fertigation has advantage in increasing crop yield, water– and nitrogen– use efficiencies^11,15,18,26–28^. For eggplant, the yield and N agronomic efficiency are augmented in the treatment of water and N via drip irrigation if compared with the treatment of soil N application and furrow irrigation (local irrigation method as control treatment)^29^. In this study, compared to no N treatment (F0), N fertigation treatments (F1-F4) raised the yield, total N uptake, WUEt and WUEs of sweet-waxy maize.

Nitrogen apparent recovery fraction (Nrf) is an index to express the percentage of nitrogen fertilizer recovery in plants^30^. In this study, there were no significant differences in Nrf among F1, F2 and F3. In the view of N rate, F3 (0.16 g N per kg soil) had the potential for a high N apparent recovery fraction. Comparatively, close to F3, F2 (0.18 g N per kg soil) had the highest nitrogen apparent recovery fraction (Table 5).

Nitrogen use efficiency (NUE) is a ratio of yield to N supply, which is regarded as an important index in evaluating fertilizer performance^31^. Hartmann *et al*.^32^ showed that NUE declines with the increase of N rates. Similarly, NUE decreased as N fertilizer rate rose in our study.

Nitrogen physiological efficiency (NPE) is an index of the plant’s ability to convert nutrients into economic yield. Peng *et al*.^33^ found that there is a positive correlation between NUE and NPE. In our study, increased N fertilizer rate also resulted in a decline in NPE.

Nitrogen agronomic efficiency (NAE) is an index to express the relationship between yield–increasing effect and per unit of applied N and it is used to evaluate the contribution of fertilizer N^34^. Our study showed that in a certain range of N rates, NAE enhanced along with the rising of N rates, but reduced when N rate was over 0.18 g N per kg soil via fertigation (F2) (Table 5), which was in consistent with the finding of Rathore *et al*.^35^ The decrease in NAE when N rate was over 0.18 g N per kg soil may result from the fact that N uptake and maize yield did not raise at the same percentage.

Root N uptake efficiency (NRE) is an index to reflect shoot plant N accumulation regarding to root contribution^36–38^. In this study, no obvious difference between F1 (0.20 g N per kg soil) and F2 (0.18 g N per kg soil) indicated that F2 can result in higher NRE with lower N rate.

Our results showed that F2 increased maize dry mass accumulation and yield, and improved total N uptake and NRE compared with F0 (no N applied) under ADI, indicated that rational nitrogen fertigation was beneficial for raising yield and N uptake of sweet-waxy maize under ADI. Moreover, the marked interaction of irrigation method and N treatment on shoot and total dry masses, WUTt, total N uptake, Nrf and NAE were found, and F2–ADI had higher shoot and total dry masses, WUEt, total N uptake, Nrf and NAE. Water and nitrogen have mutual promotion in crop growth. On one hand, suitable water supply may promote dry mass accumulation and N transport. On the other hand, appropriate N rate may maintain a suitable N concentration in the crop and enhance crop drought tolerance. Moreover, moderate N supply may increase WUE in semiarid environments^39^.

There are several reports about spatial and temporal distribution characteristics of ADI and fertigation on crops^14,39,40^. In this study, N dynamics was not observed, but crop WUE, nitrogen uptake and nitrogen use efficiencies were investigated under the combined effect of ADI and fertigation. More researches in N dynamics in responding to ADI and fertigation are needed in the future investigation.

Therefore, the yield, N uptake, WUE and NUE of sweet-waxy maize were determined not only by irrigation method, but also by N fertigation, and alternate drip irrigation increased shoot and total dry masses, nitrogen uptake, and water- and nitrogen- use efficiencies of sweet-waxy maize with nitrogen fertigation of 0.18 g N per kg soil.

## Materials and Methods

### Experimental site and materials

Pot experiment was conducted in a greenhouse in Guangxi University, Nanning, southern China (22°51′11″ N, 108°17′27″ E). The experimental soil is latosolic red soil (Orthic Acrisol, FAO-UNESCO system). The soil texture was clay soil, with a pH of 5.3, available nitrogen (N) (hydrolytic N, 1 mol l^−1^ NaOH hydrolysis) of 31.6 mg kg^−1^, available P (0.5 mol l^−1^ NaHCO_3_) of 33.3 mg kg^−1^, available K (1 mol l^−1^ neutral NH_4_OAc) of 100.3 mg kg^−1^ and soil water content at field capacity (on the mass basis) of 29.8%. Maize plant (*Zea mays* L., a local variety, Jiatian No. 11) was used for this experiment.

Plastic pots were 35 cm in upper diameter, 26 cm in bottom diameter, and 29 cm in depth. The inside of pot was sealed with a plastic sheet in the middle to separate into two even halves and prevent water exchange. Each pot was filled with 20 kg and each half contained 10 kg soil. Maize seeds were sown at the center of the pots. The primary roots were relatively evenly distributed in the two halves of the pot.

### Experimental design and implement

**Figure 4:**
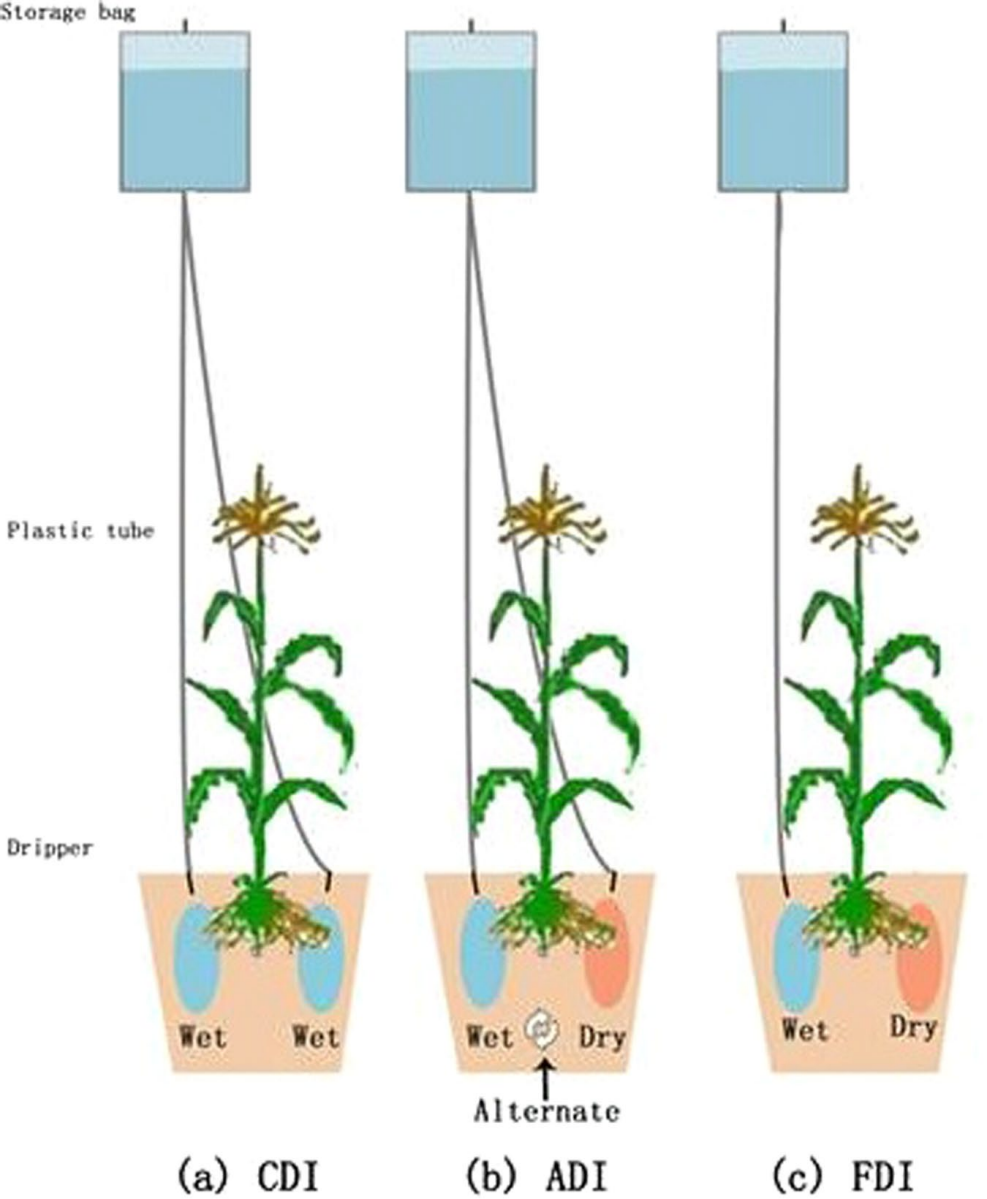
Layout of drip irrigation imitation system for three drip irrigation methods. (**a**) CDI: conventional drip irrigation (both halves of the pot simultaneously irrigated by two drippers), (**b**) ADI: alternate partial root–zone drip irrigation (both halves of the pot alternately irrigated by one dripper), (**c**) FDI: fixed partial root–zone drip irrigation (only one fixed half of the pot irrigated by one dripper).

There were three irrigation levels and five nitrogen (N) fertigation treatments in this study, totally 15 treatments (i.e. 3 × 5), and each treatment had three replicates and was conducted as a randomized block design, totally 45 pots. The 15 pots in a row were arranged in the east-west direction, and the position of the pots was randomly shifted each week. Three irrigation methods included conventional drip irrigation (CDI, both halves of the pot simultaneously irrigated by two drippers), alternate partial root-zone drip irrigation (ADI, both halves of the pot alternately irrigated by one dripper) and fixed partial root-zone drip irrigation (FDI, only one fixed half of the pot irrigated by one dripper). Before starting water control, soil moisture contents of all treatments were maintained at 70–80% of field capacity (*θ*_*f*_). Water control started at 20 days after the sowing. Weighing the pots in CDI and irrigating with tap water at interval of one day or two days depending on climate condition and plant growth status. Irrigation amount in CDI depend on soil moisture content, which was maintained within the range of 70–80% *θ*_*f*_ at the seedling stage, 75–85% *θ*_*f*_ from the jointing stage to harvest. Irrigation amount in ADI and FDI was 80% of each watering in CDI. Drip irrigation imitation system consisted of storage bag, plastic tube and dripper (Fig. 4). Storage bag was hanged in two meters high above soil surface, and water amount in the storage bag depended on the required water in each pot. One plastic tube had one flow–rate controller and one dripper at the end. Two drippers per pot were for CDI and one dripper per pot was for ADI or FDI at each irrigation event. Each dripper was kept at flow rate of 0.7 L h^−1^.

Five nitrogen (N) treatments included F0 (no N supplied), F1-F4 (0.2, 0.18, 0.16 and 0.14 g N per kg soil via fertigation, respectively). N was supplied with urea (N 46%) as topdressing via fertigation, and N fertilization dates and percentages during the growing season for different N fertigation treatments were shown in Table 6. When topdressing via fertigation, N fertilizer was dissolved in water and mixed with the needed amount of irrigation water, applied through imitated system of drip irrigation (Fig. 4). Under CDI, urea solution was evenly supplied to the two halves of the pot every topdressing. Under ADI, urea solution was alternately supplied to the two halves of the pot in consecutive topdressing, so as to maintain the two halves of the pot evenly fertilized. Under FDI, urea solution was supplied to one fixed half of the pot every topdressing. Both P and K fertilizers for all treatments were used as basal fertilizer and mixed with soils evenly at the commencement of the experiment. P_2_O_5_ (0.15 g per kg soil) and K_2_O (0.2 g per kg soil) were applied with KH_2_PO_4_ (P_2_O_5_ 52%, K_2_O 34%) and KCl (K_2_O 60%), respectively. All fertilizers were used with analytical reagents.

**Table 6:** N fertilization dates and percentages during the growing season for different N fertigation treatments. 4/30, 5/20, 5/31, 6/7, 6/17 and 6/26 were N supplied date.

N treatment	N rate (g N per kg soil)	Percentage of N applied at different growth stages (%)					
		Jointing stage		Booting stage		Maturing stage	
		4/30	5/20	5/31	6/7	6/17	6/26
F0	0	0	0	0	0	0	0
F1	0.20	15	15	20	20	15	15
F2	0.18	15	15	20	20	15	15
F3	0.16	15	15	20	20	15	15
F4	0.14	15	15	20	20	15	15

Three sprouting seeds in each pot were sown on 7 April, 2014. One plant per pot was chosen for uniformity on 26 April. Plants were harvested on 11 July.

## Measurements

### Dry mass accumulation and dry seed yield

Shoots, roots and seeds were separately harvested in each treatment. Plant materials were firstly dried at 105 °C for 30 min to inactivate enzyme in plants, and then dried at 60–70 °C to the constant mass. Finally the dry mass was weighed.

### Maize water consumption and WUE

Maize water consumption was computed using the following water balance equation in the pot^18^.

1 $$ET=I-({\theta }_{h}-{\theta }_{0})$$

where *ET* is crop evapotranspiration, *I* is total irrigation water over the growth season (liters), *θ*_*h*_ is soil water reserved at the harvest stage (liters), *θ*_0_ is soil water content at the beginning of the experiment. During the whole experiment, there was no leakage and runoff.

Water use efficiency on the basis of total dry mass (WUEt) or dry seed yield (WUEs) was calculated, respectively,

2 $${\rm{WUEt}}({\rm{kg}}\,{{\rm{m}}}^{-3})=({\rm{shoot}}\,{\rm{dry}}\,{\rm{mass}}+{\rm{root}}\,{\rm{dry}}\,{\rm{mass}})/\mathrm{total}\,{\rm{water}}\,{\rm{consumption}}$$

3 $${\rm{WUEs}}({\rm{kg}}\,{{\rm{m}}}^{-3})={\rm{Dry}}\,{\rm{seed}}\,\mathrm{yield}/\mathrm{total}\,{\rm{water}}\,{\rm{consumption}}$$

### Maize N content

Maize N content (on dry mass basis) was determined from dried ground material digested with H_2_SO_4_/H_2_O_2_ and analyzed by the Kjeldahl method^41^. Maize N uptake is the product of root, or shoot, or seed N content and the corresponding dry mass. Total N uptake is the sum of root, shoot and seed N uptakes^36^.

### Nitrogen use efficiency

Nitrogen (N) use efficiency parameters, including N apparent recovery fraction (N_rf_), N- use efficiency (NUE), N physiological efficiency (NPE), N agronomic efficiency (NAE) and root N uptake efficiency (NRE), were calculated as follows^36^,

4 $${{\rm{N}}}_{{\rm{rf}}}( \% )=({\rm{Total}}\,{\rm{N}}\,{\rm{uptake}}\,{\rm{at}}\,{{\rm{N}}}_{{\rm{t}}}-{\rm{Total}}\,{\rm{N}}\,{\rm{uptake}}\,{\rm{at}}\,{{\rm{N}}}_{0})/N\,{\rm{applied}}\,{\rm{at}}\,{{\rm{N}}}_{{\rm{t}}}\times 100$$

5 $${\rm{NUE}}({\rm{g}}\,{\rm{dry}}\,{\rm{mass}}\,{{\rm{g}}}^{-1}{\rm{N}})={\rm{Total}}\,{\rm{biomass}}\,({\rm{shoot}}+{\rm{root}}+{\rm{seed}})/\mathrm{Total}\,{\rm{N}}\,{\rm{uptake}}$$

6 $$\begin{array}{c}{\rm{NPE}}({\rm{g}}\,{\rm{dry}}\,{\rm{mass}}\,{{\rm{g}}}^{-1}{\rm{N}})\,=\,({\rm{Total}}\,{\rm{biomass}}\,{\rm{at}}\,{{\rm{N}}}_{{\rm{t}}}-{\rm{Total}}\,{\rm{biomass}}\,{\rm{at}}\,{{\rm{N}}}_{0})/\\ \phantom{\rule{10em}{0ex}}({\rm{Total}}\,{\rm{N}}\,{\rm{uptake}}\,{\rm{at}}\,{{\rm{N}}}_{{\rm{t}}}-{\rm{Total}}\,{\rm{N}}\,{\rm{uptake}}\,{\rm{at}}\,{{\rm{N}}}_{0})\end{array}$$

7 $${\rm{NAE}}({\rm{g}}\,{\rm{dry}}\,{{\rm{massg}}}^{-1}{\rm{N}})=({\rm{Total}}\,{\rm{biomass}}\,{\rm{at}}\,{{\rm{N}}}_{{\rm{t}}}-{\rm{Total}}\,{\rm{biomass}}\,{\rm{at}}\,{{\rm{N}}}_{0})/N\,{\rm{applied}}\,{\rm{at}}\,{{\rm{N}}}_{{\rm{t}}}$$

8 $${\rm{NRE}}({\rm{mg}}\,{\rm{N}}\,{{\rm{g}}}^{-1}{\rm{root}}\,{\rm{DM}})={\rm{Shoot}}\,{\rm{and}}\,{\rm{seed}}\,{\rm{N}}\,\mathrm{uptake}/\mathrm{Root}\,{\rm{dry}}\,{\rm{mass}}$$

where N_t_ is N applied treatment, N_0_ is no N added treatment.

### Statistical analyses

General linear model–univariate procedure from SPSS20.0 software was used to do analysis of variance (ANOVA). ANOVAs included main effects (irrigation method and N treatment) and their interaction. When the interaction of irrigation method and N treatment was significant, all treatments were compared using the Duncan’s multiple range tests, and the results were shown in the Figs 1, 2 and 3. Mean values for three irrigation methods or five N treatments were compared for any notable difference using the Duncan’s multiple range tests.

### Data availability

The authors declare that the data in the current manuscript are available upon request.
